# Time Trend Analysis of Tuberculosis Treatment While Using Digital Adherence Technologies—An Individual Patient Data Meta-Analysis of Eleven Projects across Ten High Tuberculosis-Burden Countries

**DOI:** 10.3390/tropicalmed7050065

**Published:** 2022-04-22

**Authors:** Liza M. de Groot, Masja Straetemans, Noriah Maraba, Lauren Jennings, Maria Tarcela Gler, Danaida Marcelo, Mirchaye Mekoro, Pieter Steenkamp, Riccardo Gavioli, Anne Spaulding, Edwin Prophete, Margarette Bury, Sayera Banu, Sonia Sultana, Baraka Onjare, Egwuma Efo, Jason Alacapa, Jens Levy, Mona Lisa L. Morales, Achilles Katamba, Aleksey Bogdanov, Kateryna Gamazina, Dzhumagulova Kumarkul, Orechova-Li Ekaterina, Adithya Cattamanchi, Amera Khan, Mirjam I. Bakker

**Affiliations:** 1 KIT Royal Tropical Institute, Global Health, 1092 AD Amsterdam, The Netherlands; liza.degroot@gmail.com (L.M.d.G.); m.straetemans@kit.nl (M.S.); 2 The Aurum Institute, Parktown, Johannesburg 2193, Gauteng, South Africa; nmaraba@auruminstitute.org; 3 Desmond Tutu Health Foundation, P.O. Box 13801, Mowbray, Cape Town 7705, Western Cape, South Africa; lauren.jennings@hiv-research.org.za; 4 De La Salle Medical and Health Sciences Institute, City of Dasmariñas Cavite 4114, Philippines; tarcelasg@yahoo.com (M.T.G.); naidamarcelo@gmail.com (D.M.); 5 Health Poverty Action, London EC1V 2NX, UK; m.mekoro@healthpovertyaction.or.ke (M.M.); p.steenkamp@healthpovertyaction.org (P.S.); r.gavioli@healthpovertyaction.org (R.G.); 6 Health Through Walls, Port-au-Prince HT 6110, Haiti; aspauld@emory.edu (A.S.); eprophete@htwinc.org (E.P.); mbury@htwinc.org (M.B.); 7 Icddr,b, GPO Box 128, Dhaka 1000, Bangladesh; sbanu@icddrb.org (S.B.); sonia.sultana@icddrb.org (S.S.); 8 KNCV Tuberculosis Foundation, 2516 AB The Hague, The Netherlands; baraka.onjare@kncvtbc.org (B.O.); egwuma.efo@kncvtbc.org (E.E.); jason.alacapa@kncvtbc.org (J.A.); jenslevy@gmail.com (J.L.); monalisa.morales@kncvtbc.org (M.L.L.M.); 9 Department of Medicine, College of Health Sciences, Makerere University, Kampala P.O. Box 7062, Uganda; amkatamba@gmail.com; 10 PATH, 01034 Kyiv, Ukraine; abogdan@path.org (A.B.); kgamazina@path.org (K.G.); 11 The Red Crescent National Society of the Kyrgyz Republic, Bishkek 720040, Kyrgyzstan; kumarkul@mail.ru (D.K.); e.orechova@redcrescent.kg (O.-L.E.); 12 School of Medicine, University of California San Francisco, San Francisco, CA 94110, USA; adithya.cattamanchi@ucsf.edu; 13 Stop TB Partnership, 1218 Geneva, Switzerland

**Keywords:** tuberculosis, digital adherence technologies, meta-analyses, implementation research, multi-country, medication adherence, mobile technologies

## Abstract

Worldwide, non-adherence to tuberculosis (TB) treatment is problematic. Digital adherence technologies (DATs) offer a person-centered approach to support and monitor treatment. We explored adherence over time while using DATs. We conducted a meta-analysis on anonymized longitudinal adherence data for drug-susceptible (DS) TB (*n* = 4515) and drug-resistant (DR) TB (*n* = 473) populations from 11 DAT projects. Using Tobit regression, we assessed adherence for six months of treatment across sex, age, project enrolment phase, DAT-type, health care facility (HCF), and project. We found that DATs recorded high levels of adherence throughout treatment: 80% to 71% of DS-TB patients had ≥90% adherence in month 1 and 6, respectively, and 73% to 75% for DR-TB patients. Adherence increased between month 1 and 2 (DS-TB and DR-TB populations), then decreased (DS-TB). Males displayed lower adherence and steeper decreases than females (DS-TB). DS-TB patients aged 15–34 years compared to those >50 years displayed steeper decreases. Adherence was correlated within HCFs and differed between projects. TB treatment adherence decreased over time and differed between subgroups, suggesting that over time, some patients are at risk for non-adherence. The real-time monitoring of medication adherence using DATs provides opportunities for health care workers to identify patients who need greater levels of adherence support.

## 1. Introduction

Worldwide, tuberculosis (TB) continues to be an enormous public health concern. It is one of the top ten causes of death in low- and middle-income countries, and until the COVID-19 pandemic, it was the leading cause of death from a single infectious agent [1]. In 2020, an estimated ten million people fell ill with TB, and although treatable, it caused 1.3 million deaths [1]. In 2014, the World Health Assembly adopted the “END TB Strategy”: by 2035, this resolution aims to reduce TB deaths by 95% and TB incidence by 90% and eliminate the catastrophic costs for patients and their families [2]. Unfortunately, the rapidly rising rates of drug resistance hampers achieving these goals [3]. One of the main causes of drug resistance is non-adherence to treatment [4]. Non-adherence could also lead to disease relapse and/or death and to an increase in treatment costs [5].

To reduce non-adherence and consequently contribute to substantial improvements in treatment outcomes, a strategy of witnessed dosing, Directly Observed Therapy (DOT), was developed [6]. While DOT allows for a direct method to verify adherence, its impact on improving treatment outcomes has been inconsistent and its implementation faces multiple limitations [7,8,9]. DOT is often poorly implemented, time-consuming, and often results in losses of income, autonomy, and privacy for patients [10]. With the global expansion of mobile phone access and improvements in network and internet connections, there has been much interest in the use of digital technologies to aid in treatment support. This interest has grown as recent restrictions and health system constraints due to the COVID-19 pandemic have further pushed countries to think of remote virtual solutions for patient management and care. Digital adherence technologies (DATs), such as 99DOTS, a phone-based technology; evriMED, a digital pillbox; and video-observed treatment (VOT), are considered to be promising tools to offer a more person-centered and time-efficient alternative to DOT for monitoring and supporting adherence [11,12]. DATs can reduce the need for travel to health care facilities (HCFs) and live, face-to-face observations by health care workers, and they may also reduce the stigma associated with community-based DOT [11,12,13,14]. Furthermore, with the ability to capture real-time adherence data, DATs potentially offer insights into patient adherence, which can be used by health care workers to offer differentiated care and more support to those who are frequently missing doses. However, to date, evidence that DATs can improve adherence and treatment outcomes is limited and has been mixed, but most studies have suggested that the technologies are at least as effective as the standard of care [7,15,16,17,18].

Adherence can be influenced by multiple and various factors. For example, the treatment phase has been shown to influence adherence; patients tend to have lower adherence in their continuation phase of treatment compared to the intensive phase [19,20]. Differences across sex have also been noted in many studies, with women generally having better adherence compared to men [21,22,23]. The influence of age on adherence is less clear; some studies found poorer adherence in younger patients, while others found poorer adherence in older patients [24,25,26,27]. Furthermore, adherence outcomes could differ based on health care workers’ skills, training, and attitudes and health system factors [27]. Whether these factors are equally relevant when using DATs remains to be determined. The existing socio-economic and gender gap in digital health and the higher prevalence of illiteracy among women in high-TB-burden countries may affect the use of a DAT [14,23,28]. Older-aged patients may also face similar disadvantages. How and when HCFs implement DATs can potentially influence adherence as well [11,27]. Thus far, there is limited research on adherence over time while using DATs and on factors that might influence adherence. This information is needed to improve DAT implementation and in turn, potentially TB treatment outcomes. TB REACH, a Stop TB Partnership initiative for innovative projects, supported the implementation of multiple DAT projects in 2018–2020 [29]. This study aims to examine the practical implication of DAT in 10 high-TB-burden countries through the analysis of TB treatment adherence over time while using DAT. Three questions were assessed: how does TB treatment adherence change over time, which factors are associated with adherence, and do time patterns in adherence differ between different subgroups?

## 2. Materials and Methods

### 2.1. Design

TB REACH supported fourteen projects that implemented DATs between 2018 and 2020 in twelve high-TB-burden countries [29]. Projects focused either on persons with drug-susceptible (DS) or drug-resistant (DR) TB, except for the project in Ukraine, which focused on both types of TB. Each project established their own inclusion and exclusion criteria and patient support and follow-up protocol based on their country’s guidelines, context, and population. The treatment adherence of patients enrolled in these DAT projects was monitored over time. Anonymized, individual, longitudinal treatment adherence data, collected by the DATs, were uploaded onto a TB REACH Dashboard, designed for monitoring and evaluating the projects. Data uploaded onto this dashboard were used in this study to perform individual patient data meta-analyses for DS-TB and DR-TB populations.

### 2.2. Study Population

Of the fourteen projects, supported by TB REACH, eleven projects in ten countries (Bangladesh, Ethiopia, Haiti, Kyrgyzstan, Namibia, the Philippines (two projects), South Africa, Tanzania, Uganda, and Ukraine) uploaded treatment adherence data to the dashboard and provided permission to be included in the analysis. Data for 6077 patients who were enrolled on a DAT were uploaded, of which 4988 patients (82.1%) were included in this study after applying exclusion criteria.

The following patients were excluded:

1. Patients with zero doses taken during all first six treatment months;
2. Patients aged fourteen years or younger;
3. Patients whose type of TB (DS or DR) differed from the type of TB the project focused on;
4. DS-TB patients who started DAT more than two weeks after they started with medication.

### 2.3. Operationalization of Variables

#### 2.3.1. Dependent Variable

*Monthly adherence*: Percentage of doses taken against doses planned per treatment month (28 days), for the first six treatment months while enrolled on a DAT. Any doses taken prior to the patient being enrolled on a DAT were not included in this analysis. A DAT sends a signal to a server when it is used, and assuming that medication was taken, this signal is registered as “dose taken”. A “dose missed” is registered when no signal is received. This, however, could be manually changed by health care workers into “dose taken”, after receiving confirmation from a patient that a dose was taken, when either the patient did not use the DAT or the signal was not sent. The data differentiate between “doses taken” that were DAT-registered versus manually registered (by health care workers). However, because of the different DAT tools and platforms that were used to capture the data, there were differences across projects in how manually registered doses were defined. This means that some projects did not use manually registered doses and for some projects, it is unknown how many of the doses taken were in fact manually registered. The total number of planned doses was calculated by extracting the DAT start date from the treatment end date, irrespective of any treatment interruptions due to adverse events. The treatment end date was defined as either the date treatment ended, or the end date of the TB REACH project. Accordingly, patients had 28 doses planned per treatment month, with possibly less doses planned in their last treatment month. The number of patients with monthly adherence data decreased from treatment month 1 to 6 due to patients who either were enrolled at a later stage of the project and thus did not complete treatment within the lifespan of the project; discontinued use of DAT (changed to DOT); died; or were “lost to follow up” (doses missing for 8 weeks and no clinical outcome available).

#### 2.3.2. Independent Variables

All independent variables were treated as categorical variables in the analyses. *Time*: treatment month 1 to 6. *Sex*: female and male; patients with unknown sex (*n* = 2) were excluded from the specific analysis on sex. *Age*: categorized into: 15–34, 35–50, and >50 years. *Enrollment period*: as patients enrolled in a project within a timeframe of one to two years, the enrollment period of a project was dichotomized into the first half and second half of the project duration. *DAT type*: 99DOTS, evriMED, and VOT. *HCF*: the facility where patients were enrolled for treatment. *Project*: eleven TB REACH DAT projects in ten countries.

### 2.4. Statistical Analysis

Meta-analyses of individual patient data were performed. All anonymized individual treatment adherence data were separated into two datasets: “DS-TB population”, which included all persons enrolled in a project that focused on DS-TB, and “DR-TB population”, which included all persons enrolled in a project that focused on DR-TB. Since the treatment of DS-TB and DR-TB differs substantially in drug combination, side effects, duration, and support/supervision of health care workers, the decision was made to analyze these populations separately. First, time trends in monthly adherence were studied to assess whether adherence increased or decreased between months. Second, differences in the average adherence over the six months of treatment (further referred to as differences in adherence) across subgroups were studied to assess if one group (e.g., females) showed higher or lower adherence than another group (e.g., males). Third, differences in time trends in monthly adherence across subgroups were studied, i.e., did one group (e.g., patients on 99DOTS) show a steeper decrease or increase over time than another group (e.g., patients on evriMED)? All analyses were performed in STATA version 15.0.

#### 2.4.1. Descriptive Analysis

Monthly adherence percentages were categorized into five groups: 0–5%, >5 to ≤50%, >50 to <90%, 90 to <100%, and 100%; these categories were used within the TB REACH framework. Frequency tables and belonging stack bar graphs were made to study the proportions of patients in the adherence categories over the six months and to assess patterns in adherence. Tables and graphs were made for all patients, whereafter they were disaggregated by sex, age, enrollment period, DAT type, and project.

#### 2.4.2. Regression Analysis

Three level tobit regression explanatory analyses were performed in which repeated measures were clustered within individuals, and individuals were clustered within HCFs. We expected health care workers’ engagement to play a role in treatment adherence. Patients attached to a facility where a highly engaged health care worker provided care were expected to be more similar in treatment adherence than patients from the same project across multiple facilities.

Monthly adherence held strong floor (0%) and ceiling (100%) effects which a tobit regression analysis took into account, as well as the non-normal distribution of the outcome variable due to these floor and ceiling effects [30]. This made it possible to analyze monthly adherence as a continuous variable [31]. To indicate the degree of correlation of the monthly treatment adherence of patients within the same HCF, the intraclass correlation coefficient (ICC) from an HCF intercept-only model was calculated. In the case in which the ICC was higher than 0.1, a correction for clustering within HCF was considered necessary. Furthermore, all analyses were corrected for the number of planned doses per patient, because patients could have had fewer doses planned in their last treatment month.

To assess how monthly adherence changed over time, we built a base model with monthly adherence (in percentages), the six time points (in months), and the number of planned doses. The time reference category was constantly changed to analyze the differences in monthly adherence between two consecutive months. To assess which factors were associated with adherence, the independent variables (sex, age, and enrollment period) were simultaneously added to the base model. DAT type and project were separately added to the base model to account for collinearity. An independent variable was considered to be a factor associated with adherence whenever its regression coefficient was significant (*p* < 0.05). To assess if time patterns in monthly adherence differed between subgroups, interaction terms were made between time and the independent variables (sex, age, enrollment period, DAT type, and project) in bivariate models. The variables were considered effect modifiers when the interaction term was significant (*p* < 0.05). When analyzing differences between DAT types within the DS-TB population, patients on VOT were excluded (*n* = 41), because only Haiti implemented VOT. The following categories were used as reference categories: females, patients aged > 50 years, first half (enrollment period), 99DOTS (in DS-TB population), and evriMED (DR-TB population). The 99DOTS technology for the DS-TB population and evriMED for DR-TB population were used as references as they were the most used DAT among their respective populations. All projects were used as a reference category once, to see whether there were significant differences across projects. During the second and third analyses, on differences in adherence and differences in time trends in monthly adherence across subgroups, month 1 was set as the time reference category.

#### 2.4.3. Sensitivity Analyses

Sensitivity analyses were carried out, in which the manually registered doses taken, which were the results of health care workers’ actions, were excluded from the total amount of doses taken to see how much these affected the results. All analyses as described above were performed again.

## 3. Results

An overview of the eleven DAT projects and their enrolled patients is provided in Table 1, showing diversity in the number of patients included across projects, ranging from 22 (Namibia) to 1351 (Uganda), as well as differences in target group and inclusion criteria. Eight of the eleven projects focused on DS-TB, two projects focused on DR-TB, and only the project in Ukraine focused on both DS-TB and DR-TB. Furthermore, six projects implemented 99DOTS, two VOT, two evriMED pill boxes, and one project implemented both VOT and evriMED pill boxes. Table 2 provides an overview of the study population, disaggregated by TB population (DS-TB and DR-TB). More male patients (63.6%) were included than female patients (36.4%). Additionally, most patients (48.5%) were aged 15 to 34 years. Around one-fifth of all “doses taken” were manually registered by health care workers.

### 3.1. DS-TB Population

**Figure 1:**
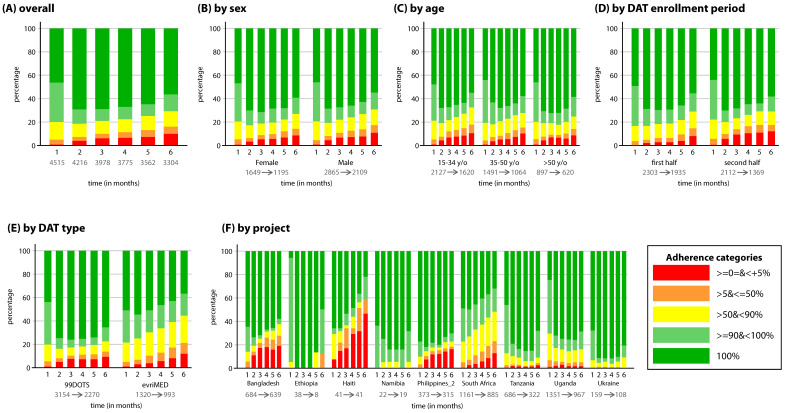
Frequency graphs of TB treatment adherence categories for DS-TB population over the (first) six treatment months. The proportion of patients belonging to a certain adherence category is depicted on the *y*-axis, and the six treatment months are depicted on the *x*-axis. (**A**) Monthly adherence for all patients. (**B**) Monthly adherence split up by sex. (**C**) Monthly adherence split up by age category. (**D**) Monthly adherence split up by DAT enrollment period. (**E**) Monthly adherence split up by DAT type. (**F**) Monthly adherence split up by project. Underneath each subgroup two numbers are placed; these resemble the sample size at month one (before arrow) and month six (after arrow). Number of patients at each month is given in the overall graph. DAT = digital adherence technology; y/o = years old.

The overall average adherence rate among patients with DS-TB varied between 80% to 90% across the projects (Table 1). An increase in the proportion of patients with 100% adherence between month 1 and month 2 was seen, followed by a continual decline in the proportion of patients with 100% and ≥90 to <100% adherence (Figure 1A), indicating an increase in adherence at first, followed by a decrease. Tobit analysis confirmed these findings: see the first and second row in Table 3. Moreover, the proportion of DS-TB patients with ≥90% adherence (dark and light green charts) declined from 79.9% in month 1 to 70.8% in month 6, signifying a decrease in adherence over time, although the proportion of people with 100% adherence was higher at month 6 (56.7%) compared to month 1 (46.6%). The pure ICC of the HCF intercept-only model was 0.24, indicating that the monthly adherence of patients within one HCF was correlated, and correction for this correlation is needed.

The proportion of patients with ≥90% adherence was slightly higher amongst females than males (79.7% vs. 76.5%), suggesting that males had lower adherence overall in comparison to females (Figure 1B). Tobit analyses confirmed lower adherence in males (*p* < 0.01) and a steeper decrease during every month (*p* < 0.05) in comparison to females (Table 3, row three and four). Patients > 50 years old showed higher adherence than the two younger groups (Figure 1C), but this was not statistically significant (Table 3, row three). Tobit analysis indicated a steeper decrease in adherence for patients aged 15–34 years old compared to patients aged >50 years old (Table 3, row four). Relatively more patients who enrolled on DAT during the second half of projects’ enrollment periods showed either very poor (0–5%) or 100% adherence, compared to patients who enrolled during the first half of enrollment (Figure 1D). Tobit analyses found lower adherence for patients in the second half of enrollment (*p* < 0.01), and a steeper decrease at month 2, month 4, and month 6 (*p* < 0.05), compared to patients in the first half of enrollment (Table 3, row three and four). The comparison of DAT types showed a larger proportion of patients with 0–5% and 100% adherence in 99DOTS patients compared to patients on evriMED (Figure 1E). Moreover, the frequencies of adherence categories stayed more or less stable in patients on 99DOTS at month 2, month 3, month 4, and month 5. Tobit analyses did not find statistically significant differences in adherence, but they did find a steeper decrease in adherence in evriMED patients compared to 99DOTS during every month (*p* < 0.01) (Table 3, row four). Great diversity in adherence and in time trends in monthly adherence development across projects is seen in Figure 1F and was seen in the tobit analyses (*p* < 0.01) (Table 3).

### 3.2. DR-TB Population

**Figure 2:**
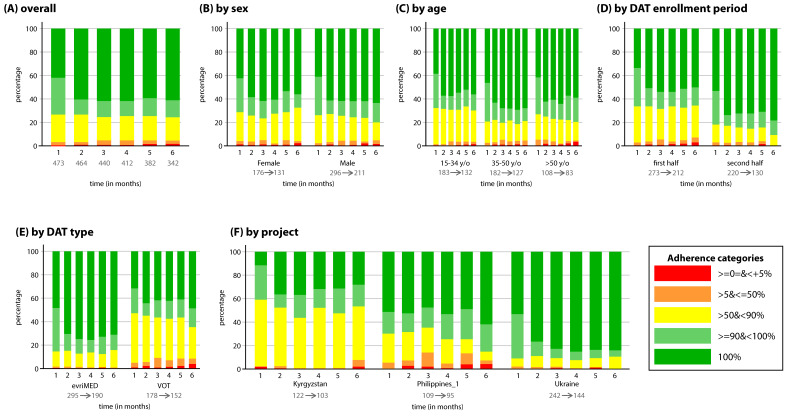
Frequency graphs of TB treatment adherence categories for DR-TB population over the (first) six treatment months. The proportion of patients belonging to a certain adherence category is depicted on the *y*-axis, and the six treatment months are depicted on the *x*-axis. (**A**) Monthly adherence for all patients. (**B**) Monthly adherence split up by sex. (**C**) Monthly adherence split up by age category. (**D**) Monthly adherence split up by DAT enrollment period. (**E**) Monthly adherence split up by DAT type. (**F**) Monthly adherence split up by project. Underneath each subgroup two numbers are placed; these resemble the sample size at month one (before arrow) and month six (after arrow). Number of patients at each month is given in the overall graph. DAT = digital adherence technology; VOT = video-observed therapy; y/o = years old.

The overall average adherence rate among patients with DR-TB varied between 80% and 87% across the projects (Table 1). An increase in the proportion of patients with 100% adherence was seen between month 1 and month 2 (Figure 2A). In the following months, the proportions of all adherence categories stayed more or less stable, except for the 0–5% category, which increased over time. Tobit analysis found an increase in adherence between month 1 and month 2 (*p* < 0.01), and month 2 and month 3 (*p* > 0.05), followed by a decreasing pattern (*p* > 0.05) (Table 3, row two). Additionally, the proportion of patients with ≥90% adherence barely differed between month 1 (73.2%) and month 6 (75.4%). The pure ICC was 0.27, which indicated that the monthly adherence of patients within one HCF was correlated, and correction for this was needed.

No clear differences were seen in monthly adherence between males and females (Figure 2B) and different age categories (Figure 2C, Table 3). The proportion of patients with 100% adherence was larger in the group of patients who enrolled in the second half of a project, compared to patients who enrolled in the first half. Additionally, the proportion of patients with >50%–<90% adherence was smaller in the second group than in the first group (Figure 2D). Tobit analyses also found higher adherence for patients who enrolled later in a project (*p* < 0.01) (Table 3, row three). Furthermore, a steeper increase in adherence at month 6 for those enrolled in the second half of a project was found (*p* < 0.01). The proportion of patients with <90% adherence was larger in patients on VOT in comparison to patients on evriMED (see Figure 2E), indicating that patients on VOT had lower registered adherence. Tobit analyses showed consistent findings with lower adherence (*p* < 0.01), and a steeper decrease in adherence at month 3, month 4, month 5, and month 6 (*p* < 0.01), for patients on VOT compared to patients on evriMED. Clear differences in adherence were seen between projects (Figure 2F). Tobit analyses confirmed these differences both in adherence (*p* < 0.05) and in time trends in monthly adherence (*p* < 0.05).

### 3.3. Sensitivity Analyses

Additional patients were excluded from the sensitivity analyses when excluding the manually registered doses taken, as more patients had zero doses. Therefore, the DS-TB population reduced from 4515 to 4399 patients and the DR-TB population from 473 to 408. The outcomes of the tobit regression analyses changed slightly. The same subgroups showed lower adherence and/or a steeper decrease in adherence; however, the level of significance and/or treatment month in which significant differences were seen changed. The overall time pattern when excluding the manually registered doses in the DS-TB population changed: the initial increase in adherence from month 1 to 2 disappeared and changed into a continuous, statistically significant decrease. Table S1 in the Supplementary Materials shows the findings of the tobit regression analyses when excluding the manually registered doses.

## 4. Discussion

Our analysis is the first to combine multiple DAT projects in high-burden countries to better understand factors associated with adherence. The overall average adherence among patients with DS-TB varied between 80% and 90%, and among patients with DR-TB, between 80% and 87% across the projects. We found that the DATs indicated high levels of adherence throughout treatment for most patients: 80% to 71% of DS-TB patients had ≥90% adherence in month 1 and 6, respectively, and 73% to 75% for DR-TB patients. The adherence rates found in this study are at least similar to those found in previous research, although some studies showed higher rates [32].

Statistically significant differences in adherence and time trends in monthly adherence were found across subgroups. Male DS-TB patients showed lower adherence and a steeper decrease over time compared to females; this difference was not statistically significant in DR-TB patients. The youngest DS-TB patients (15–34 y/o) showed a steeper decrease in adherence than the oldest group (>50 y/o). DS-TB patients on evriMED showed a steeper decrease than DS-TB patients on 99DOTS. Meanwhile, DR-TB patients on VOT showed lower adherence and a steeper decrease than patients on evriMED.

Our analyses focused on time trends in TB treatment adherence per treatment month across multiple projects, instead of dichotomizing treatment time in intensive and continuation phases. A possible explanation for the ‘increase followed by decrease’ found in this study could lie in the digital aspect of a DAT. Previous research found that TB patients may be more comfortable with using a digital treatment monitoring app over time, meaning that they know how to use the technology better [33]. Perhaps this led to better adherence at first, whereafter the “usual” observed adherence pattern occurred: a decrease in adherence from the intensive phase to the continuation phase, as demonstrated by other studies [34,35,36]. The sensitivity analysis, however, did not confirm this pattern and showed a continues decrease in adherence without the increase between month 1 and 2. These trends need further exploration when more data become available. Decreasing adherence over time might be caused by the fact that some patients may still suffer from side effects, which may result in treatment discontinuation, or patients may experience fewer symptoms during the continuation phase and misinterpret this as being cured [34,35]. Consequently, patients may be less eager to take medication. Alternatively, this adherence pattern could also be attributed to disinterest by patients in engaging with the DAT to record that the dose was taken. Decreased engagement with digital health tools over time has been previously documented in other studies [37,38]; however, disengagement with the tool might not necessarily reflect suboptimal medication intake [39,40]. A study on whether or not the use of 99DOTS accurately represents if a dose was taken found that 99DOTS tends to underestimate adherence. Using urine samples to determine if patients took their medication, the study found that patients often took medication without using the tool [41].

In our analyses, males exhibited lower adherence than females, despite the inequality in access to digital health tools across sex [28]. This is consistent with findings from other TB treatment adherence studies [20,21,22,42,43,44,45]. Potential reasons for this could be that males are more likely to engage in risk behaviors (i.e., alcohol abuse, drug use, and smoking) that are often associated with lower adherence [25,46,47,48,49]. Moreover, women were found to be more efficient and effective than men at utilizing digital tools to monitor adherence [33]. Across all projects, there was a steeper decrease in adherence in patients aged 15–34 than patients aged > 50; consensus on the effect of age on adherence was not found in the literature [43,50,51,52,53].

Differences in adherence across DAT types were also found in our analyses. The DAT tools included in our projects have different functionalities and require different levels of effort in engagement by the patients to record dose intake. A potential advantage of the evriMED digital pill box is its automated feature that whenever the pillbox is opened, a signal is automatically sent to a server [54]. This can be perceived as less complex and time-consuming compared to 99DOTS or VOT [54]. However, 99DOTS offers a potentially less stigmatizing opportunity for patients to take their medication discretely and privately with the use of a cell-phone rather than a pill box [54]. This could potentially explain the lower adherence observed in our projects using evriMED, as stigma can negatively influence treatment adherence [55]. VOT, on the other hand, offers high confidence that drugs are taken (compared to evriMED and 99DOTS) but also requires more effort from the patients. Patients require access to a smartphone, tablet, or computer with internet access to record and upload a video of themselves ingesting medication [54,56,57]. This might explain the steep decrease in adherence as over time, patients may find the process burdensome and time-consuming with continued use. Except for Kyrgyzstan, all projects focused on one DAT. Therefore, it is challenging to make a proper comparison in adherence across DAT types; it is uncertain whether it is the DAT specifics or projects’ characteristics that were associated with adherence.

Our analyses also found differences in reported adherence depending on when during project implementation a patient was enrolled on treatment. Each project reported challenges at the beginning of implementing DATs, both for patients as well as health care workers, which were resolved along the way [56]. This could explain higher adherence in DR-TB patients who enrolled in the second half of a project compared to patients in the first half. However, this does not explain the lower adherence found in DS-TB patients who started DAT in the second half of a project compared to patients who enrolled in the first half. A potential explanation for this observance could be due the diminishment of conditions and factors that may have facilitated initial implementation over time [58]. For example, many projects reported frequent changes in program staff and health care workers who may not have received as comprehensive training on how to use DAT as their counterparts did during the initial rollout of the tools.

The adherence of individuals was shown to be clustered within HCFs. Training on using DATs to manage patients could have varied between HCFs. Moreover, staff at different HCFs may have different skill levels in building rapport and engaging with patients. Numerous studies emphasized the importance of a satisfying relationship between patients and health care workers to achieve good adherence [19,59,60,61,62]. This critical component of patient care cannot be substituted through the use of a DAT alone. Understandably, some health care workers are more prone to motivate patients or follow-up after missed doses. The latter could also depend on workload; rural areas with frequent internet disruptions could require more follow-up by HCWs, which can take time.

All projects faced implementation problems, affecting the ability to document adherence minimally or extensively. For example, Haiti suffered from civil unrest and prison riots, and almost all projects faced network and internet connection difficulties, yet treatments continued [56]. Next to this, diverse in-and exclusion criteria were applied: some projects only included patients who showed good adherence in the past or only patients with a (smart)phone, whereas other projects enrolled all new TB-patients on DAT [56]. During analyses, patients without any doses taken, reflecting 0% adherence, were excluded. These could have led to selection bias, leading to higher or lower adherence. Great diversity was seen in action protocols after “dose missed”: some protocols stated a call should be made the same day, and others after three doses were missed [56]. Moreover, not all projects changed a missed dose into a dose taken if a follow-up call revealed that the patient did take a dose that was not captured by the DAT. Additionally, differences in DAT usage were found, e.g., evriMED patients from Kyrgyzstan received extra support from a “public helper”, a relative/neighbor, who reminded patients to take their medication and observed patients taking their medication [56].

The large sample size of this study gives a more precise estimation of the time trends, and differences found in the reported regression analysis made it possible to analyze adherence as a continuous variable and analyze time trends per treatment month. This offers richer perspectives on adherence behavior than a summarized overall adherence rate with a single number (all doses taken vs. all doses planned during full treatment time) or compared to dichotomous adherence measurements (above or below a certain threshold), which have been studied in previous research [63,64,65]. Moreover, the time trend analyses conducted in this study gives valuable insights into the complex, dynamic, and longitudinal patterns related to medication adherence in long-term therapies [64]. Since tobit analysis is a form of mixed model analysis, it was possible to correct for clustering within groups (i.e., individual and HCF), which appeared to be necessary.

However, two assumptions of tobit analysis were not met. First, tobit analysis should be used when upper and lower limits are reached because of censoring; patients actually score higher or lower than the measurement tool allows [30,31]. Yet, in this study, patients did not score higher or lower than 100% and 0%. Second, while tobit analysis assumes the distribution between floor and ceiling is normal, it does allow for a non-normal distribution of the outcome variable, as was the case for our data. As a result of this, the regression coefficients found using the tobit analyses were not clinically plausible. Yet, *p*-values belonging to these regression coefficients were used to determine whether certain patterns or differences were statistically significant. In our large sample size, this might result in small changes in the level of adherence becoming statistically significant. Despite these factors, we chose this type of regression analysis because it was the best available method to analyze the longitudinal adherence data, which was also confirmed using the model fit indicators.

It is important to note that our analyses assumed when a “dose taken” was registered on a DAT platform, either automatically or manually, that patients were adherent to their treatment. However, these registrations are only proxies for medication intake, except for VOT. While the literature suggests that these may be valid methods to measure treatment adherence [66,67,68,69], recent research has questioned how accurately the tools can reflect whether or not a dose was taken [41]. Additionally, as previously described, the number of doses taken in this study was a combination of “dose taken” as registered by DAT and those manually registered by health care workers. However, because of the different DAT tools and platforms that were used to capture the data, there were differences across projects in whether and how manuals were defined. The sensitivity analyses did not reveal many differences when excluding the manually registered doses taken. The only remarkable difference was seen within the DS-TB population, where the initial increase in adherence from month 1 to 2 disappeared and changed into a continuous, statistically significant decrease. Additionally, the data presented reflect individual patient adherence data downloaded from DAT platform servers. Although these data were not compared to individual patient dosing histories and therefore may contain some errors, these are unlikely to impact the adherence trends reported here.

## 5. Conclusions

Our meta-analyses indicate that adherence decreases over time and that some patients are more prone to non-adherence than others. Real-time monitoring of medication adherence using DATs provides opportunities for health care workers to identify patients who need a greater level of adherence support. While our study was not able to determine if the observed adherence patterns were due to non-adherence or disengagement with using the digital tool, these findings are similar to other studies that have looked at adherence and emphasize that differentiated support may be needed to keep these patients on treatment (over time). Moreover, our study also highlights that project implementation (when and where) may be associated with patient adherence, and thus, efforts to assess and offer training to health care workers on how to optimally use DATs to continue to engage patients should be continued. Additional analyses on data collected by DATs should be conducted to determine if patterns of adherence can determine which patients are in need of additional treatment support and to explore if and why these differ among DS-TB and DR-TB patients. More research is also needed to see if these tools can be used to assist TB patients with co-morbidities such as diabetes or HIV with their different treatments. Additionally, in the absence of shorter treatment regimens, future research should explore the challenges and benefits associated with the technical aspects of DAT and with its deployment amongst TB patients and health care workers to improve DAT implementation.

## Figures and Tables

**Table 1:** Overview of the eleven DAT projects and their enrolled patients.

	Bangladesh	Ethiopia	Haiti	Kyrgyzstan		Namibia	Philippines_1	Philippines_2	South Africa	Tanzania	Uganda	Ukraine	
**DAT ^1^ type**	99DOTS	99DOTS	VOT ^2^	evriMed	VOT	99DOTS	VOT	99DOTS	evriMED	99DOTS	99DOTS	evriMED	evriMED
**N total**	719	44	77	54	85	24	110	396	1258	976	1535	540	258
**N study**	684	38	41	53	69	22	109	373	1161	686	1351	159	242
**Target group**													
Age	≥8	≥16	≥18	18–65		≥16	≥13	≥15	≥2	>15	≥19	≥18	
Type of TB	DS-TB	DS-TB	DS-TB	DR-TB		DS-TB	DR-TB	DS-TB	DS-TB	DS-TB	DS-TB	DS-TB	DR-TB
Additional characteristics	private patients from Dhaka	(semi-) nomadic/agro- pastoralists	prisoners	continuation phase from Bishkek and Chui-region		semi-mobile hunters and gatherers	semi-urban	urban poor, elderly, HIV+	N/A	rural miners	N/A	from Mykolayiv and Odesa oblasts	
**Enrollment dates**													
Start	10-4-2019	29-3-2019	9-3-2019	11-1-2019		9-4-2019	27-12-2018	6-12-2018	1-5-2019	25-2-2019	10-1-2019	13-2-2019	
End	28-7-2020	27-2-2020	21-2-2020	28-12-2019		13-3-2020	14-12-2019	16-3-2020	16-10-2020	30-6-2020	31-12-2019	11-11-2019	
**Inclusion criteria** Additional to: informed consent mentally, physically, and psycho-socially able	no MDR-TB live closely to Dhaka access to mobile phone	residence in mobile phone coverage area network coverage	able to operate mobile phone and/or tablet	≥2 weeks ambulant treatment ≥80% adherence first 2 weeks (hospitalized) internet access ability to use electronic device		residence in mobile phone coverage area network coverage	≥2 weeks on treatment access to mobile phone	newly diagnosed access to mobile phone	≤2 weeks on treatment access to mobile phone	access to mobile phone with minimum balance	access to mobile phone	TB doctor decided who to offer the box at first only patients who showed good adherence in past; later, also newer patients; patients enrolled after hospitalization	
**Sex** *n* (%)													
Female	275 (40.2)	12 (31.6)	0	18 (34.0)	34 (49.3)	16 (72.7)	38 (34.9)	101 (27.1)	413 (35.6)	260 (37.9)	506 (37.5)	66 (41.5)	86 (35.5)
Male	409 (59.8)	26 (68.4)	41 (100)	35 (66.0)	34 (49.3)	6 (27.3)	71 (65.1)	271 (72.7)	748 (64.4)	426 (62.1)	845 (62.6)	93 (58.5)	156 (64.5)
Unknown					1 (1.4)			1 (0.3)					
**Age** median (IQR) ^3^	31 (22;45)	29 (23;41)	31 (27;37)	47 (33;60)	29 (24;40)	25.5 (20;34)	30 (39;51)	32 (25;47)	37 (29;46)	43 (32;56)	36 (27;46)	38 (32;46)	39 (31;47)
**Age categories** *n* (%) 15–34 y/o ^4^ 35–50 y/o >50 y/o	391 (57.2) 164 (24.0) 129 (18.9)	22 (57.9) 8 (21.1) 8 (21.1)	29 (70.3) 9 (22.0) 3 (7.3)	14 (26.4) 14 (26.4) 25 (47.2)	43 (62.3) 19 (27.5) 7 (10.1)	17 (77.3) 4 (18.2) 1 (4.6)	44 (40.4) 37 (33.9) 28 (25.7)	208 (55.8) 96 (25.7) 69 (18.5)	553 (47.6) 406 (35.0) 202 (17.4)	222 (32.4) 241 (35.1) 223 (32.5)	623 (46.1) 494 (36.6) 234 (17.3)	62 (39.0) 69 (43.4) 28 (17.6)	82 (33.9) 112 (46.3) 48 (19.8)
**Enrollment period** *n* (%) First half Second half	360 (52.6) 324 (47.4)	8 (21.1) 30 (78.9)	19 (46.3) 22 (53.7)	45 (85.9) 8 (15.1)	61 (88.4) 8 (11.6)	9 (40.9) 13 (59.1)	53 (48.6) 56 (51.4)	247 (66.2) 126 (37.8)	787 (67.8) 374 (32.2)	359 (52.3) 327 (47.7)	462 (34.2) 889 (65.8)	52 (32.7) 107 (67.3)	114 (47.1) 128 (52.9)
**HCF ^5^** *n*	5	2	5 (prisons)	10	11	1	6	3	9	11	18	14	16
**Time points** *n* (%) Month 1 Month 2 Month 3 Month 4 Month 5 Month 6	684 (100) 671 (98) 662 (97) 653 (95) 644 (94) 639 (93)	38 (100) 28 (74) 24 (63) 15 (39) 15 (39) 8 (21)	41 (100) 41 (100) 41 (100) 41 (100) 41 (100) 41 (100)	53 (100) 53 (100) 52 (98) 52 (98) 49 (92) 46 (87)	69 (100) 68 (99) 67 (97) 67 (97) 63(91) 57(83)	22 (100) 20 (91) 19 (86) 19 (86) 19 (86) 19 (86)	109 (100) 108 (99) 105 (96) 103 (84) 98 (80) 95 (78)	373 (100) 364 (98) 355 (95) 336 (90) 326 (87) 315 (84)	1161 (100) 1090 (94) 1003 (86) 947 (82) 909 (78) 885 (76)	686 (100) 565 (82) 481 (70) 428 (62) 373 (52) 322 (47)	1351 (100) 1286 (95) 1248 (92) 1204 (89) 1112 (82) 967 (72)	159 (100) 151 (95) 145 (91) 132 (83) 123 (77) 108 (68)	242 (100) 235 (97) 216 (89) 190 (79) 172 (71) 144 (60)
**Doses taken manually registered** *n* (%)	3135 (3.6)	3069 (95.1)	0 (0)	9721 (57.2)		3209 (1.9)	0 (0)	6080 (12.2)	0 (0)	28186 (39.4)	76891 (43.0)	2903 (13.6)	4059 (13.1)
**Overall average adherence** (planned/taken)	90%	81%	80%	80%	81%	81%	82%	84%	84%	88%	86%	87%	87%

^1^ DAT = digital adherence technology; ^2^ VOT = video-observed therapy; ^3^ IQR = interquartile range; ^4^ y/o = years old; ^5^ HCF = health care facility.

**Table 2:** Overview of study population, disaggregated by type of TB population.

		DS-TB ^1^ Population (*n* = 4515)	DR-TB ^2^ Population (*n* = 473)
Projects *n* (%)	Bangladesh Ethiopia Haiti Kyrgyzstan Namibia Philippines_1 Philippines_2 South Africa Tanzania Uganda Ukraine	684 (15.1) 38 (0.8) 41 (0.9) 22 (0.5) 373 (8.3) 1161 (25.7) 686 (15.2) 1351 (29.9) 159 (3.5)	122 (25.8) 109 (23.0) 242 (51.2)
DAT ^3^ type *n* (%)	99DOTS evriMED VOT ^4^	2468 (64.5) 1320 (34.5) 41 (1.1)	295 (62.4) 178 (37.6)
Sex *n* (%)	Female Male Unknown	1389 (36.3) 2439 (63.7) 1 (0.0)	176 (37.2) 296 (62.6) 1 (0.2)
Age median (IQR) ^5^		35 (27;46)	38 (30;49)
Age categories *n* (%)	15–34 y/o ^6^ 35–50 y/o >50 y/o	1905 (49.8) 1250 (32.7) 674 (17.6)	183 (38.7) 182 (38.5) 108 (22.8)
Enrollment period *n* (%)	First half Second half	1944 (50.8) 1885 (49.2)	273 (57.7) 200 (42.3)
HCF ^7^ *n*		58	35
Time points *n* (%)	Month 1 Month 2 Month 3 Month 4 Month 5 Month 6	3829 (100) 3651 (95) 3497 (91) 3347 (87) 3189 (83) 2982 (78)	473 (100) 464 (98) 440 (93) 412 (87) 382 (81) 342 (72)
Doses taken manually registered *n* (%)		120,324 (21.6)	13,780 (21.9)

^1^ DS-TB = drug-sensitive tuberculosis; ^2^ DR-TB= drug-resistant TB; ^3^ DAT = digital adherence technology; ^4^ VOT = video-observed therapy; ^5^ IQR = interquartile range; ^6^ y/o = years old; ^7^ HCF = health care facility.

**Table 3:** Findings of tobit regression analyses; time trend in TB treatment adherence, factors associated with adherence, and differences in time trends across subgroups.

		DS-TB ^I^ Population (*n* = 4515)	DR-TB ^II^ Population (*n* = 473)
Overall statement		Increase followed by decrease	Increase followed by decrease
Time trend between months	1–2	↑ **	↑ **
	2–3	↓ **	↑
	3–4	↓ **	↓
	4–5	↓ **	↓
	5–6	↓ **	↓
Factors	Sex	Males − **	Males −
	Age (years)	15–34 − 35–50 −	15–34 + 35–50 +
	DAT ^III^ start date	Second half − **	Second half + **
	DAT type	evriMED +	VOT ^IV^ − *
	Project	+/− *	+/− *
Time patterns between subgroups	Time * sex	Males ↘ * ^3,4,5,6^	+/ −
	Time * age	15–34↘ * ^3,4,5,6^	+/ −
	Time * Enrollment period	Second half ↘ * ^2,4,6^	Second half ↗ ** ^6^
	Time * DAT type	evriMED ↘ ** ^all^	VOT ↘ ** ^3,4,5,6^
	Time * project	+/− *	+/− *

**Between months**: ↑ = increase; ↓ = decrease. **Factors**: + = category had higher adherence than reference category; − = category had lower adherence than reference category; +/− = differences in adherence between projects. **Time patterns between sub-groups**: ↗ = subgroup had a steeper increase in adherence over time than reference group; ↘ = subgroup had a steeper decrease in adherence over time than reference group; +/− = different directions of regression coefficients between months, or between projects. **Reference categories**: females (sex), >50 (age), first half (enrollment period), 99DOTS in DS-TB, evriMED in DR-TB (DAT), projects (changed). * *p* < 0.05, ** *p* < 0.01, 2,3,4,5,6 = month at which difference is significant. Light gray when not significant. ^I^ DS TB = drug-sensitive tuberculosis; ^II^ DR TB = drug-resistant TB; ^III^ DAT = digital adherence technology; ^IV^ VOT = video-observed therapy.

## Data Availability

The data presented in this study are available on request from the corresponding author. The data are not publicly available, as the study datasets are property of the National TB Control Programs and the organizations of the projects included in the meta-analyses.

## Supplementary Materials

The following supporting information can be downloaded at: https://www.mdpi.com/article/10.3390/tropicalmed7050065/s1, Table S1: Findings of tobit regression analyses; time trend in TB treatment adherence, differences in adherence across subgroups and differences in time trends across subgroups excluding the manually registered doses.
