# Supplementary material for: Time Trend Analysis of Tuberculosis Treatment While Using Digital Adherence Technologies—An Individual Patient Data Meta-Analysis of Eleven Projects across Ten High Tuberculosis-Burden Countries
Source: Trop Med Infect Dis. 2022 Apr 22;7(5):65. doi: 10.3390/tropicalmed7050065 (PMC9145978; doi:10.3390/tropicalmed7050065)
Supplement: Supplementary file 1 [file tropicalmed-07-00065-s001.zip › tropicalmed-1548392-supplementary.pdf]

Tropical Medicine and Infectious Disease

Supplementary material of Time Trend Analysis of Tuberculosis Treatment While Using Digital Adherence Technologies—An Individual Patient Data Meta-Analysis of Eleven Projects across Ten High-Tuberculosis-Burden Countries

Table S1. Findings of tobit regression analyses; time trend in TB treatment adherence, differences in adherence across subgroups and differences in time trends across subgroups excluding the manually registered doses.

		DS-TB Population (n = 3829)	DR-TB Population (n = 473)
**Overall statement**		Decrease	Fluctuating
**Between months**	*1-2*	↓**	↑
	2-3	↓**	↓
	3-4	↓**	↓
	4-5	↓**	↑
	5-6	↓**	↓
**Factors**	*Sex*	Males –**	Males –
	Age (years)	15-34 –<br>35-50 –	15-34 +<br>35-50 –
	Enrollment period	Second half —**	Second half +*
	DAT type	evriMED +**	VOT –**
	Project	+/–*	+/–*
**Effect modifiers**	*Time*sex*	Males ↘*2,5,6	+/–
	*Time*age*	15-34↘*3,4	+/–
	*Time*enrollment period*	Second half ↘*3,4	Second half ↗*6
	*Time*DAT type*	evriMED ↘**3,4↗**6	VOT ↘*3,4,5
	*Time*project*	+/–*	+/–*

Between months: ↑ = increase; ↓ = decrease.
Factors: + = subgroup had higher adherence than reference group; – = subgroup had lower adherence than reference group' +/– = differences in adherence between projects.
Effect modifiers: ↗ = subgroup had a steeper increase in adherence over time than reference group; ↘ = subgroup had a steeper decrease in adherence over time than reference group; +/– = different directions of regression coefficients between months, or between projects.
Reference categories: females (sex), >50 (age), first half (DAT start date), 99DOTS in DS-TB, evriMED in DR-TB (DAT), projects (changed).
*p<0.05, **p<0.01, 2,3,4,5,6 = month at which difference is significant. Light grey when not significant. Differences with the main analyses are colored orange. [I] DS TB= drug-sensitive tuberculosis; [II] DR TB= drug-resistant TB; [III] DAT=digital adherence technology; [IV] VOT=video-observed therapy; [V] HCF=health care facility.
